# Extensive research into MERS-CoV to ascertain its current status, epidemiological context, and potential in the future

**DOI:** 10.1097/JS9.0000000000000227

**Published:** 2023-03-03

**Authors:** Shopnil Akash, Nobendu Mukerjee, Farjana I. Aovi, Swastika Maitra, Athanasiosis Alexiou

**Affiliations:** a Department of Pharmacy, Faculty of Allied Health Sciences, Daffodil International University, Ashulia, Dhaka, Bangladesh; b Department of Microbiology, West Bengal State University; c Department of Microbiology, Adamas University, Kolkata, West Bengal, India; d Department of Health Sciences, Novel Global Community Educational Foundation, Australia; e Department of Science and Engineering, Novel Global Community Educational Foundation, Ha-bersham, Australia; f AFNP Med, Wien, Austria

*Dear Editor*,

The Middle East respiratory syndrome coronavirus, often known as MERS-CoV, is a new emerging beta coronavirus that belongs to lineage C. About 80% of all human cases of this virus have been reported to Saudi Arabia, and more than 2600 confirmed cases have been diagnosed in humans with at least 1000 fatalities globally since it was first detected in 2012^1^. Since MERS-CoV was discovered for the first time in 2012, it has been claimed that the death rate among individuals with laboratory-confirmed infection is roughly 30–40%^2^. It may trigger severe complications of pulmonary disease in people. Most MERS-CoV infections are asymptomatic or produce minor symptoms such as fever, chest tightness, and coughing. On the other hand, severe infections are more likely to develop in older persons with concurrent conditions such as cancer, diabetes, or renal and lung disorders. Other symptoms, including pneumonia and diarrhea, have also been recorded^2^.

According to a recent disease outbreak statement issued by the WHO, there have been four laboratory-confirmed cases of MERS-CoV documented from three different areas inside the Kingdom of Saudi Arabia. WHO revealed in April 2022 that the Kingdom of Saudi Arabia had been the source of six prior MERS-CoV infections, including four fatalities, between August 2021 and February 2022. The most current epidemic includes three cases that occurred between 29 December 2021, and the end of October 2022. Two cases originated in Riyadh, while the other came from Gassim and Makka Al Mukarramah. Real-time PCR confirmed the instances^1^.

The MERS-CoV genome is estimated to have 10 open reading frames (ORFs) and that the first 5′-three-fourths of the sequence encode for RdRp (ORF1a and ORF1b)^3^. Using −1 ribosomal frame shifting, these two ORFs produce the polyproteins pp1a and pp1b, which are further cleaved into 16 nonstructural proteins (nsps)^4^. All of the structural proteins – spike (S), envelop (E), membrane (M), and nucleocapsid (N) – are encoded by the final three ORFs, which are located downstream of ORF1b (N). S protein facilitates viral entry by interacting with dipeptidyl peptidase 4. Since the MERS-CoV S protein is very variable, it has been a focus of phylogenetic research and the development of therapeutics^5^,^6^.

Besides, research has found that the molecular epidemiology of MERS-CoV is similar to the initial human cases, which were first diagnosed in 2012. The current molecular features of the MERS-CoV virus are ~99% similar to the sequences observed in comparison to the first human cases, and the level of pathogenicity is also reported as the same^7^.

The MERS-CoV virus is a zoonotic pathogen, meaning it may be spread from animals to humans. Although the precise transmission mode is unknown, research has found that people get sick via contact with diseased dromedary camels. Several Middle Eastern, African, and South Asian Member States have confirmed the presence of MERS-CoV in dromedary camels^8^. Latest investigations in human populations with occupational contact with dromedary camels in several Member States show that there is also zoonotic transmission happening in several African Member States, despite only a small number of human cases being recorded outside of the Middle East.

Transmission between humans is feasible and has already happened, most often between intimate friends and family members, particularly in medical settings. Many others might be affected, such as members of the patient’s immediate family, home, and healthcare providers. Most of the disease has spread via hospitals in Saudi Arabia, the United Arab Emirates, and the Republic of Korea. There is no evidence of persistent human-to-human transmission outside healthcare settings^9^,^10^.

Diagnostics are crucial in the early identification and management of any pathogenic disease. They also offer a more comprehensive knowledge of the epidemiological data and risk factors for MERS-CoV^11^. Measurement and identification of MERS-CoV infestations in the laboratory have often comprised molecular detection of MERS-CoV RNA; assays to identify a humoral response to prior MERS-CoV infection among humans; and MERS-CoV antigen detection. Molecular diagnostics can identify nucleic acids obtained from MERS-CoV in clinical samples collected from the respiratory tract, serum, and stool. Because traditional nucleic acid-based tests require the use of specialist molecular instruments and techniques, they are unsuitable for point-of-care testing or assessment at the bedside. This is one of the most significant challenges associated with these tests^12^,^13^.

The WHO has provided guidelines for treating severe respiratory illnesses likely caused by the MERS-CoV virus. Nevertheless, there is currently no antiviral therapy that is specifically indicated for the treatment of MERS-CoV infection^14^. So, developing innovative medicine is highly needed to fight against MERS-CoV, and global scientific communities should invest in finding a suitable vaccine for long-term effectiveness against this deadly pathogen.

## Ethical approval

Not applicable/not required. This is correspondent does not require any human or animal subjects to acquire such approval.

## Sources of funding

This research did not receive any specific grant from funding agencies in the public, commercial, or not-for-profit sectors.

## Author contribution

S.A., N.M.: conceptualization, study design, and writing. S.A., F.I.A., S.M., and N.M.: writing and analyzed the data. F.I.A., S.M., N.M., and A.A.: editing and reviewing.

## Conflicts of interest disclosure

Authors declare no conflict of interest exists.

## Research registration unique identifying number (UIN)

1. Name of the registry: not required.
2. Unique identifying number or registration ID: not required.
3. Hyperlink to your specific registration (must be publicly accessible and will be checked): not required.

## Guarantor

Shopnil Akash (corresponding author) takes full responsibility for the work and/or the conduct of the study, has access to the data, and controls the decision to publish.

## Data availability statement

Extensive research into MERS-CoV is needed to ascertain its current status, epidemiological context, and potential in the future.
